# Midlife insomnia and subsequent mortality: the Hordaland health study

**DOI:** 10.1186/1471-2458-14-720

**Published:** 2014-07-15

**Authors:** Børge Sivertsen, Ståle Pallesen, Nick Glozier, Bjørn Bjorvatn, Paula Salo, Grethe S Tell, Reidun Ursin, Simon Øverland

**Affiliations:** 1 Division of Mental Health, Norwegian Institute of Public Health, Bergen, Norway; 2 Uni Health, Uni Research, Bergen, Norway; 3 Department of Psychiatry, Helse Fonna HF, Haugesund, Norway; 4 Department of Psychosocial Science, University of Bergen, Bergen, Norway; 5 Norwegian Competence Center for Sleep Disorders, Haukeland University Hospital, Bergen, Norway; 6 Brain and Mind Research Institute, Sydney Medical School, The University of Sydney, Sydney, Australia; 7 Department of Global Public Health and Primary Care, University of Bergen, Bergen, Norway; 8 Finnish Institute of Occupational Health, Helsinki, Finland; 9 Department of Psychology, University of Turku, Turku, Finland; 10 Department of Biomedicine, University of Bergen, Bergen, Norway

**Keywords:** Insomnia, Risk factor, Mortality, Sleep duration, Sleep medication

## Abstract

**Background:**

Previous research suggests a possible link between insomnia and mortality, but findings are mixed and well-controlled studies are lacking. The aim of the current study was to examine the effect of insomnia in middle age on all-cause mortality.

**Methods:**

Using a cohort design with 13-15 years follow-up, mortality registry data were linked to health information obtained during 1997-99, as part of the community-based Hordaland Health Study (HUSK), in Western Norway. 6,236 participants aged 40–45 provided baseline information on self- reported insomnia using the Karolinska Sleep Questionnaire Scale (defined according to the 5^th^ edition of the Diagnostic and Statistical Manual of Mental Disorders (DSM-V), sociodemographic factors, health behaviors, shift/night-work, obstructive sleep apnea symptoms, sleep duration, sleep medication use, anxiety, depression, as well as a range of somatic diagnoses and symptoms. Height, weight and blood pressure were measured. Information on mortality was obtained from the Norwegian Cause of Death Registry.

**Results:**

Insomnia was reported by 5.6% (349/6236) at baseline and a significant predictor of all-cause-mortality (hazard ratio [HR] = 2.74 [95% CI:1.75-4.30]). Adjusting for all confounders did not attenuate the effect (HR = 3.34 [95% CI:1.67-6.69]). Stratifying by gender, the effect was especially strong in men (HR = 4.72 [95% CI:2.48-9.03]); but also significant in women (adjusted HR = 1.96 [95% CI:1.04-3.67]). The mortality risk among participants with both insomnia and short sleep duration (<6.5 hours) was particularly high, whereas insomnia in combination with normal/greater sleep duration was not associated with mortality.

**Conclusions:**

Insomnia was associated with a three-fold risk of mortality over 13-15 years follow-up. The risk appeared even higher in males or when insomnia was combined with short sleep duration, although such unadjusted subgroup analyses should be interpreted with caution. Establishing prevention strategies and low-threshold interventions should consequently be a prioritized task for public health policy.

## Background

Numerous studies have investigated the association between sleep duration and mortality [1-3], but evidence linking insomnia to mortality is less clear. Whereas some studies have reported a significant association between insomnia and mortality [4-8], others have not [9-16]. To further complicate matters, some studies have even found a *protective* effect of insomnia on mortality [17,18].

The inconsistent results could reflect heterogeneous study designs, inclusion of different covariates and different operationalizations of insomnia. For example, a recent study of older adults in Taiwan found that the association between insomnia and mortality depended on the insomnia definition [19]. Some studies have investigated insomnia without accounting for sleep duration or use of hypnotic medication, both factors themselves associated with increased mortality, closely linked to insomnia and therefore potential confounders. For example, Finn et al. [4] found a hazard ratio (HR) of insomnia for mortality of 2.1, but neither sleep duration nor use of hypnotics were taken into account. Similarly, a study by Vgontzas et al. [7] found a significant effect of insomnia on mortality, but without adjusting for use of hypnotic medication.

Another possible explanation for the inconsistent results is that the association between insomnia and mortality is constrained to certain groups. The Penn State Cohort demonstrated that insomnia was associated with mortality in men sleeping less than 6 hours, whereas no significant effects of insomnia were found for men with longer sleep duration, or for women [7]. Mallon et al. [6] also found a gender specific effect, although the only other study controlling for additional sleep covariates did not [5].

The ambiguous results in the existing literature concerning the insomnia–mortality association therefore require further examination, taking into account possible subgroup differences, important confounders and correlates of insomnia such as hypnotic use and sleep duration. In addition, insomnia should be defined according to formal diagnostic criteria. Based on these considerations, the aims of the current study were: 1) to examine the independent effect of insomnia defined according to the insomnia criteria found in the 5^th^ edition of the Diagnostic and Statistical Manual of Mental Disorders (DSM-V) [20] on mortality risk after adjusting for several confounders, including sleep covariates, in a community-based sample from Norway (the Hordaland Health Study), and 2) to examine whether the potential effect of insomnia on mortality depended on gender and sleep duration.

## Methods

### Study population and data material

The Hordaland Health Study (HUSK) comprises a joint epidemiological research project carried out by the Norwegian Health Screening Service in collaboration with the University of Bergen. The base population for the study reported herein included all 29,400 individuals in the Hordaland County, Western Norway born 1953–57, aged 40–45 years. Baseline data were collected by questionnaires and a brief clinical examination, which included measurements of height, weight and blood pressure. A total of 18,581 (8,598 men and 9,983 women) completed a baseline questionnaire and met for the clinical examination, yielding a participation rate of 63% (57% for men and 70% for women). At this visit, all participants were randomly given one of two additional questionnaires. Only one of these questionnaires included detailed information on sleep. Of the 8,896 individuals receiving this second questionnaire, 6,236 (70%) provided valid responses on the sleep variables relevant for this study. The HUSK study was conducted from 1997 to 1999, with the exception of July, due to summer vacation. Previous findings from both the HUSK study and a similar large population-based study in Norway found no monthly or seasonal variations in neither sleep duration, subjective sleep need, nor insomnia prevalence [21,22].

### Measures

#### Outcome

The primary outcome of the present study was mortality from any cause as registered by the Norwegian Cause of Death Registry. This registry is kept by Statistics Norway and includes information on cause of death for all deceased persons registered as residents in Norway at the time of death.

#### Exposure

The DSM-V criteria for insomnia include difficulty falling asleep or difficulty maintaining sleep for a period of 3 months or more. In addition, it is a prerequisite that the sleep disturbance impairs daily functioning [20]. In this study, insomnia was assessed using the Karolinska Sleep Questionnaire [23]. The measurement of the insomnia diagnosis was based upon four items, each rated along a five-point scale (“never”, “rarely (a few times per year)”, “sometimes (a few times per month)”, “mostly (several times a week)” or “always”). Subjects were categorized as having insomnia if they reported problems with sleep onset, sleep maintenance, or early morning awakening (or a combination) “several times a week” or “always” over the past three months, in addition to reporting daytime tiredness/sleepiness mostly/several days per week. Although the exposure was measured long before the latest revision of the DSM, the questions used were more in line with the updated DSM-V criteria than previous DSM-versions. The Karolinska Sleep Questionnaire has been shown to have good psychometric properties [24], and was recently validated against clinical diagnosis of insomnia in a Norwegian sample [25].

To examine if there was a potential effect beyond the diagnostic cut-off value (insomnia versus not insomnia), we also used a continuous measure of insomnia as an independent variable. This variable was created by adding the scores on the 3 items assessing nocturnal insomnia symptoms (score ranging from 0-4: sleep onset problems, sleep maintenance problems, or early morning awakenings) as well as the single daytime tiredness/sleepiness item. The sum score ranged from 0-16.

#### Other participant characteristics

Level of education was reported in four categories ranging from less than seven years of schooling up to at least 4 years of higher education at college/university. We also collected data on marital/cohabitant status (dichotomized into living alone or with a partner), smoking (number of daily smoked cigarettes) and weekly level of exercise: [1] no or easy physical activity 1 hr/week, [2] moderate physical activity 1 to 2 hrs/week, or [3] hard physical activity more than 2 hrs/week. Alcohol consumption was categorized according to weekly number of self-reported alcohol units (none, 1-2 units/week, 3-4 units/week, or ≥ 5 units/week). Body mass index (BMI) was calculated by dividing weight (kg) by squared height (m^2^). The lifestyle behaviors and BMI were included as confounders due to previous studies linking them both to insomnia [26] and mortality [27].

Questions on somatic diagnoses were framed the following way: “Do you have or have you had (one or more of the following)”: myocardial infarction, stroke, diabetes, or angina. Endorsement of these alternatives were considered self-reported diagnoses positive, coded dichotomously to indicate presence or not of that condition.

Subjects were also asked about the frequency of 17 common bodily symptoms related to different organ systems in accordance with the 10^th^ edition of the International Classification of Disorders (ICD-10) Research Criteria for F45 Somatoform Disorders [28] on a five point (0-4) Likert scale labeled: “almost never, rarely, sometimes, often and almost always”. The items were summed and the composite score reflects “organ system symptoms” that is used as a continuous variable, with increasing levels reflecting higher symptom load.

Musculoskeletal pain was assessed by the following introductory question: “Have you during the last year experienced pain and/or stiffness in muscles or joints lasting for at least three consecutive months”? If yes, the participants were asked to indicate the location(s) from the following list (“yes” or “no”): neck, shoulder, elbow, hands/wrist, chest/abdomen, upper back, lower back, hips, knees, ankles or feet. Based on the responses we then constructed a dichotomous variable, where those who reported pain from any of these locations where defined as having musculoskeletal pain.

Symptoms of current non-vegetative anxiety and depression symptoms were measured using the Hospital Anxiety and Depression Scale (HADS) [29], a self-report questionnaire comprising 14 items, each with a four-point response alternatives. Seven items reflect anxiety symptoms (HADS-A) and seven reflect symptoms of depression (HADS-D). In the analyses the HADS-scores were used as continuous variables. The HADS has been found to perform well in assessing severity of anxiety and depression in both hospital settings (for which it was first designed), but also in primary care patients and the general population [30]. A cut-off score of 8 gives the best balance between sensitivity and specificity at about 0.80 for depression according to DSM-III and IV, ICD-8 and 9 criteria, and is supported as a case-identifying symptom level in general practice [31].

Symptoms of obstructive sleep apnea (OSA) were estimated using three items from the Karolinska Sleep Questionnaire [23]. These items were used to identify individuals at-risk for OSA based on their own or their partner’s reports on “snoring” and “breathing cessation” during sleep. In addition to the requirement of reporting both of these core symptoms either “sometimes (several times a month)”, “often (several times a week)” or “always”, participants were only classified as having symptoms of OSA if they also were “tired or sleepy at work or during spare time”, “sometimes”, “often” or “always”. This operationalization has also been used in a previous study based on the same data examining the effect of OSA on work disability [32]. A similar definition in line with the Hawaiian Sleep Questionnaire (the Apnea Score) has previously been shown to identify 100% of the cases with severe sleep apnea (Apnea-Hypopnea Index [AHI] > 40) and 75% of all sleep apnea cases with AHI > 5, and shown an overall predictive accuracy of 88% for AHI > 10 [33].

Use of hypnotic medication was assessed in two ways. First, participants were asked if they had used hypnotic medication on a daily or nearly daily basis over the last year, and for how many months they had used them. For the current analyses, a continuous variable comprising months of use was employed. Second, participants answered an open-ended question were they provided the name of all medications they took the day before baseline assessment. These were coded according to the Anatomical Therapeutic Chemical (ATC) classification system [34], and for the current study all mediations in the ATC–subgroup N05C (hypnotics and sedatives) were included (barbiturates, aldehydes and derivatives, benzodiazepine derivatives, piperidinedione derivatives, benzodiazepine related drugs, melatonin receptor agonists, and other hypnotics and sedatives).

Sleep duration was defined as time in bed (calculated from self-reported bedtime and rise time) minus self-reported sleep latency. For analyses purposes, sleep duration was split into 5 groups based on percentiles, according to the following cut-off points: 5.5 hours = 5th percentile; 6.5 hours = 25th percentile; 7.5 hours = 75th percentile, 8.5 hours = 95th percentile. We also dichotomized sleep duration into “short sleepers” (less than 6.5 hours) and “normal/long sleepers” (6.5 hours or more). Sleep duration was assessed separately for workdays and weekends; in the present article we focus only on sleep duration on workdays; as such sleep data are more consistent [22].

### Statistical analysis and models

STATA/SE 13.1 was used for all analyses. Independent sample t-tests and χ^2^-tests were used to examine demographic and clinical differences between participants with insomnia and good sleepers. Cox proportional hazards models were computed to assess the effect of insomnia on all-cause mortality, both crude and adjusting for each covariate separately. The following variables were included as potential confounders: age, gender, education, health behaviors, body mass index, somatic diagnoses, somatic symptoms, musculoskeletal pain, mental disorders, obstructive sleep apnea, sleep medication use, and sleep duration (entered as a 5-class categorical variable). We also tested for interactions between insomnia and sex, and insomnia and sleep duration, by entering the product of these variables in separate blocks. A final multivariate model including all covariates was also employed. In addition to using insomnia as a dichotomous variable, we also tested the effect of the continuous total insomnia score on mortality. Participants were followed from the date of participation in HUSK (1997-1999) to their death or end of follow-up (December 31, 2012), at which point they were censored (range of follow-up: 13 to 15 years). Results are presented as hazard ratios (HR) with 95 percent confidence intervals (95% CI). We evaluated the proportional hazard assumption by inspecting the log minus log plots stratified on the level for each covariate and found no major deviation from a proportional hazard. Although no detailed analyses on cause-specific deaths were conducted due to the limited sample size, simple cross-tabulations were examined. As a sensitivity analysis, we repeated all analyses excluding participants who died during the first two years of the follow-up. This was done to reduce a possible bias if the measure of insomnia was capturing a severe illness resulting in both sleep disturbance and a high likelihood of imminent death. We tested for multicollinearity by inspecting tolerance values and Variance Inflation Factors (VIFs) using the ‘collin’ command in STATA, and all values were well within the recommended limits [35]. Visual inspection of plots showed no sign of a curvilinear association between insomnia symptoms (continuous score) and mortality. Missing values were handled using listwise deletion.

### Ethics

The study protocol was approved by the Regional Committee for Medical Research Ethics of Western Norway and approved by the Norwegian Data Inspectorate. Written consent was obtained from all subjects included in this study.

## Results

### Sample characteristics

Baseline demographic and clinical characteristics are presented in Table 1. The prevalence of insomnia was 5.6%. Insomnia was more prevalent among women, current smokers, persons with low education, and among those with low physical activity. Insomnia was also positively associated with BMI, symptoms of anxiety, depression and OSA, self-reported angina and number of somatic symptoms and musculoskeletal pain, as well as use of sleep medication and short sleep duration. Insomnia was unrelated to alcohol consumption, blood pressure, and to reported diabetes, myocardial infarction and stroke.

**Table 1:** **Baseline demographic and clinical characteristics according to insomnia status in the Hordaland Health Study, Norway, 1997-1999**^**#**^

**Characteristics**	**No insomnia**	**Insomnia**	***P*-Value**
N, %	5887, 94.4%	349, 5.6%	
Age^§^	42.6 (1.5)	42.6 (1.5)	.67
Women	61.3%	71.8%	<.001
Education			.014
Compulsory	16.9%	23.0%	
High school	45.8%	42.2%	
College/university	37.3%	34.9%	
Number of daily smoked cigarettes^§^	11.0 (6.7)	12.7 (7.1)	<.001
Alcohol consumption†			.77
0 units/week	8.3%	9.4%	
1-2 units/week	32.6%	32.2%	
3-4 units/week	31.5%	29.5%	
≥ 5 units/week	27.6%	28.9%	
Physical activity			<.001
No or easy	13.5%	22.7%	
Moderate	42.5%	38.8%	
Heavy	44.0%	38.5%	
Shift/night-work	24.2%	24.1%	.98
Body-mass index (BMI)^§^	25.2 (3.8)	25.6 (4.5)	.030
Systolic blood pressure^§^	126.3 (14.5)	125.9 (14.4)	.64
Myocardial infarction	0.3%	0.3%	.60
Angina	0.4%	1.4%	.013
Stroke	0.2%	0.6%	.22
Diabetes	0.8%	1.1%	.36
Somatic symptoms^§^	10.2 (7.1)	21.2 (9.1)	<.001
Musculoskeletal pain	13.7%	43.4%	<.001
Anxiety score^§^	4.0 (2.9)	8.9 (3.9)	<.001
Depression score ^§^	2.7 (2.5)	6.3 (3.7)	<.001
Obstructive sleep apnea symptoms	4.6%	14.7%	<.001
Daily sleep medications use (months)^§^	0.1 (2.0)	1.7 (5.8)	<.001
Sleep medications (ATC-subgroup N05C)	0.1%	5.2%	<.001
Sleep duration			<.001
< 5.5 hours	2.9%	13.5%	
5.5-6.5 hours	15.3%	26.3%	
6.5-7.5 hours	48.0%	36.5%	
7.5-8.5 hours	28.8%	15.7%	
> 8.5 hours	5.1%	8.0%	

^#^Missing data were overall all low and ranged from 0% (age, gender, BMI, systolic blood pressure) to 10.7% (sleep duration).

^†^1 unit equals approximately 12 g ethanol.

^§^Data presented as mean (SD).

### The effect of insomnia on all-cause mortality

**Figure 1:**
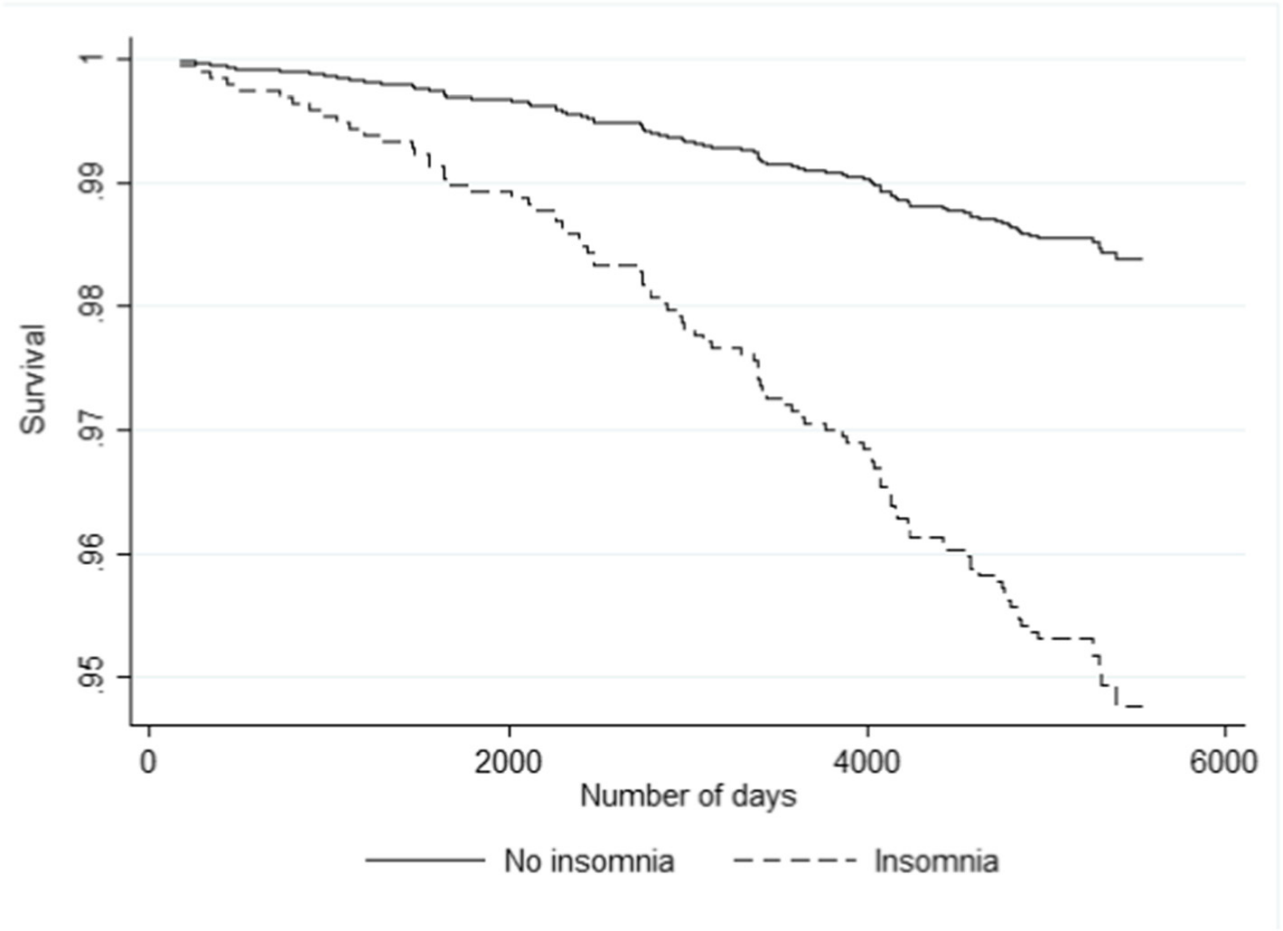
Kaplan-Meier survival curves by insomnia status (fully adjusted analyses) in the Hordaland Health Study (1997-1999).

During the follow-up period from 1997-1999 through 2012, 160/6233 (2.6%) persons died, of which 93/3858 (2.4%) were female and 67/2377 (2.8%) were male. In the crude analyses, insomnia was associated with an almost three-fold increase in mortality (hazards ratio (HR) = 2.74; 95% CI: 1.75–4.30; Figure 1 and Table 2). Including the confounders as adjustment variables, one at a time, only slightly changed the effect estimates. In the fully adjusted model, insomnia was even more strongly associated with mortality than in the crude analysis (HR = 3.34; 95% CI: 1.67–6.69).

**Table 2:** Crude and covariate-adjusted^* ^**hazard ratios of mortality risk associated with insomnia, during 14 years follow-up of the Hordaland Health Study (1997-1999)**

	**Hazard ratio**	**95% confidence intervals**
Adjustment variables:		
Crude (insomnia only)	2.74	1.75 – 4.30
Age, gender and education	2.82	1.79 – 4.44
Health behaviors^a^	2.59	1.75 – 4.30
Shift/night-work	2.97	1.87 - 4.72
Body mass index	2.71	1.73 – 4.26
Somatic diagnosis^b^	2.67	1.70 – 4.20
Somatic symptoms	2.74	1.67 – 4.54
Musculoskeletal pain	2.88	1.80 – 4.59
Mental disorders^c^	2.59	1.54 – 4.35
Obstructive sleep apnea symptoms	2.86	1.82 – 4.51
Sleep medications	2.94	1.84 – 4.70
Sleep duration	2.20	1.26 – 3.86
Fully adjusted analyses	3.34	1.67 – 6.69

^*^Covariates are adjusted for one by one. Fully adjusted analyses include all covariates.

^a^Smoking, alcohol, and physical exercise.

^b^Systolic blood pressure, myocardial infarction, angina, stroke, diabetes.

^c^Anxiety and depression symptom load.

**Figure 2:**
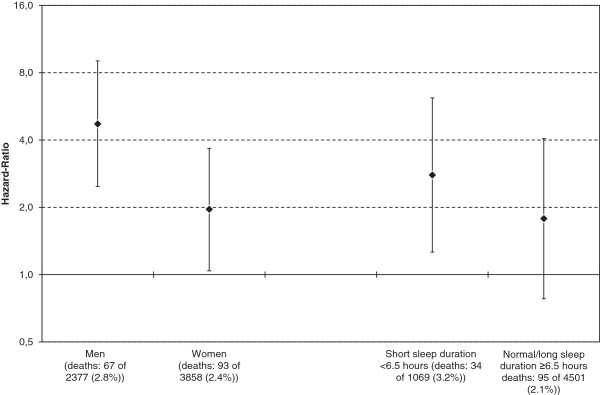
**Unadjusted hazard-ratios of mortality risk associated with insomnia, stratified by sex and sleep duration during 13-15 years follow-up of the Hordaland Health Study (1997-1999).** Error bars represent 95% confidence intervals. Note that y-axis is on a logarithmic scale.

When stratifying the analyses by gender we found that male insomniacs had almost 4 times higher risk of mortality compared to male good sleepers (crude HR = 4.72; 95% CI: 2.48–9.03; Figure 2), whereas the corresponding effect for women was HR = 1.96 (95% CI: 1.04–3.67). The interaction between insomnia and gender was statistically significant with a stronger effect of insomnia for males compared to females (*P* = 0.050).

The mortality risk was higher in insomniacs sleeping less than 6.5 hours (crude HR = 2.79; 95% CI: 1.26–6.17; Figure 2) compared those sleeping 6.5 hours or more. The association between insomnia with normal or greater sleep duration and mortality was not significant (crude HR = 1.78; 95% CI: 0.78–4.06). However the interaction between insomnia and sleep duration was not statistically significant (*P* = 0.44).

As a sensitivity analyses, we repeated the analyses after excluding deaths occurring in the first 2 years of follow-up. This resulted in 8 deaths being omitted, still the associations remained practically identical: crude HR = 2.76 (95%CI: 1.74-4.38) and adj. HR = 3.29 (95% CI: 1.60–6.78).

We also repeated the analyses using the continuous insomnia score as exposure variable. These analyses showed that the crude HR was 1.08 (95% CI: 1.02-1.15), equal to an 8.3% increase in mortality rate for every increasing value of the insomnia score (range 0-16). The fully adjusted HR was 1.10 (95% CI: 1.01-1.20), indicating a 10.1% increase in mortality rate.

### Cause of death

Although the low numbers of deaths in the current study prevented detailed analyses of cause of death, crude analyses showed no clear pattern of specific causes being more prevalent in the insomnia vs. the non-insomnia group. The most common cause of death among insomniacs was cancer (48%) followed by CVD (9.5%), as was also the case in the non-insomnia group.

## Discussion

The aim of this study was to investigate the effect of insomnia on mortality in a middle-age population, after taking into account the effect on mortality of many possible confounders including sleep duration, use of sleep medications and symptoms of OSA. In short, insomnia increased the risk of death 3-fold in the fully adjusted analyses. In exploratory analyses the effect of insomnia was appeared especially strong for men, and among insomniacs who also reported short sleep duration.

The link between sleep and mortality has been extensively studied over the last decades. Whereas the evidence of harmful effects of short and long sleep duration (see [1-3] for review) and use of hypnotics [36] is ample, the literature on any association between insomnia and mortality has been much more inconsistent. An important issue when studying any consequence of sleep, is to differentiate between distinct types of sleep problems. For example, although there is a significant overlap between those who report insomnia and those with short sleep duration [37], it is also important to distinguish between these two groups. In addition to the diagnostic criteria of difficulty falling asleep and maintaining asleep, associated with daytime impairment or distress, a diagnosis of insomnia also requires that the sleep disturbance must occur despite adequate opportunity and circumstances for sleep [38]. As such, voluntary or enforced (e.g. post-partum) sleep loss, characteristic for behaviorally induced insufficient sleep syndrome, should not be misclassified as insomnia, although such patients normally report sound sleep [39]. The distinction between sleep deprivation and insomnia is important when studying their respective consequences. For example, whereas sleep deprivation consistently has been linked to reduced neuropsychological performance [40] although with marked intra-individual variability, the effect of insomnia on this outcome is less clear, and at best more subtle and qualitatively different from sleep loss [41]. Furthermore, patients with advanced or delayed sleep phase syndrome normally complain of early morning awakening and sleep onset difficulties, respectively, and it is not uncommon that studies misattribute such symptoms to insomnia rather than to a circadian rhythm sleep disorder [42].

We were able to identify 17 studies [4-19,43] investigating the effect of insomnia on mortality, of which 5 studies found a harmful effect [4-8]. Among these five, only two studies controlled for both sleep duration, use of hypnotic medication and OSA, in addition to other somatic and psychiatric (depression) confounders [5,6]. So far no study has investigated the relationship between DSM-V based insomnia criteria and mortality. Our multivariable adjusted findings show that insomnia increased the risk of mortality 3.3 times compared to good sleepers, an effect somewhat larger than found by Hublin et al. [44]. In agreement with the latter study, gender stratified analyses in the current study suggested that this association was stronger in men: Male insomniacs were 4.7 times more likely to die than non-insomniac males in the crude analyses, while a lower, but still significant effect was observed for women (HR = 2.0). We also found a possible, although not statistically significant, interaction effect of insomnia and short sleep duration on mortality. Participants reporting both insomnia and sleeping less than 6.5 hours had 2.8 times higher risk of dying during follow-up than non-insomniacs. A similar effect of the deleterious combination of short sleep duration and insomnia was also observed by Vgontzas et al. [7] in a polysomnographic study of insomniacs.

The findings from the current study suggest that the insomnia-mortality association may be confined to subgroups: males and short sleepers. This may be part of the explanation why many previous studies have not found an independent effect of insomnia on mortality. Inconsistent operationalization of insomnia is another possible reason for the mixed findings. In the current study, we used a definition of insomnia, which resembles both The Research Diagnostic Criteria (RDC) for insomnia [38] as well as the DSM-V criteria [45]. A similar definition was used in the Hublin et al. study in which a somewhat smaller effect was observed. In contrast, some of the studies failing to find an effect of insomnia have relied on less-than-optimal definitions, often resorting to a single item, to assess insomnia. However, both Lack et al. [12] and Philips et al. [14] used well-defined operationalizations of insomnia, but nevertheless found no association between insomnia and deaths. Thus, operationalization issues may not explain all the observed discrepancies. To sum up, although the literature on insomnia predicting death is mixed, the current study clearly shows a strong link between well-characterized insomnia and mortality even after adjusting for confounders.

The big question is then: Why does insomnia predict premature death? Individual covariates in the current study only partly explained the association, and multiple adjustment actually increased the hazard ratio. For example, we found that adjusting for smoking, alcohol use and physical exercise reduced the effect of insomnia on mortality only to some extent (from HR = 2.7 to HR = 2.6). To our surprise, adjusting for depression and somatic diagnoses (hypertension, myocardial infarction, angina, stroke, or diabetes) did not indicate that these comorbid conditions are important pathways. However, the lack of mediation by somatic diagnosis may relate to the study sample being relatively young, and thereby healthier than older adults. With cancer being by far the most common cause in this middle age group many such somatic diagnoses and risk factors would be unlikely to be a mediator. The gender differences in the current study led us to speculate that there might be certain occupational aspects linking insomnia to mortality, but we found no evidence of insomnia being linked to accidents in hazardous occupations [46], although were limited by the limited number of cause specific outcomes. As such, the question as to why insomnia may increase the risk of death remains open to further examination, especially in terms of exploring more specific the causes of deaths.

There are important methodological considerations to the present study that should be noted. First, the restriction of the sample to participants aged 40-45 limits the generalizability of the findings to other age groups. Second, insomnia was assessed by self-report rather than based on a clinical diagnosis. However, the questionnaire used in our study, the Karolinska Sleep Questionnaire, is relatively analogous to the criteria for insomnia as specified by the DSM-V [20], and the prevalence found in the present study (5.6%) is identical to a previous report using a stringent DSM-IV definition of insomnia [47], suggesting a reasonable estimate of insomnia disorder prevalence in the current study. However, we only measured insomnia on one occasion, and as sleep problems commonly fluctuate over time, it would be better to have repeated measurements over a long time period in order to identify chronic insomniacs. An important addition to the DSM-IV and DSM-V from previous versions was the inclusion of a clinical significance criterion to almost half of all the categories (including insomnia), which required that symptoms cause clinically significant distress or impairment in social, occupational, or other important areas of functioning. A similar impact on family, social or occupational life is also required in the latest ICD-10 criteria for insomnia. Still, the definition of insomnia in the current study operationalized the daytime criterion through self-reported tiredness or sleepiness only, and there are other domains of daily functioning that could be affected beyond these items.

There are also other limitations of the present study related to sleep measurement issues: First, the operationalization of OSA was based on a brief self-report questionnaire. Although the prevalence estimate found in the present study (8.4%) is similar to that found in other epidemiological studies in similar age groups [48-52], the use of self-reported symptoms to measure OSA remains problematic because persons are often unaware of their behavior during sleep. However, the Karolinska Sleep Questionnaire is based on sleep problems also reported by the person’s spouse or co-habitant and thus attempts to improve the validity of reported symptoms. Another source of confounding is that both the OSA and insomnia operationalizations included the same item assessing reduced daytime functioning (daytime/sleepiness). Second, the measure of sleep duration did not include reports of wake time after sleep onset (WASO). As such, there is a risk that we may have overestimated sleep duration, especially in persons with maintenance and early morning awakening insomnia. Third, self-reported use of sleep medications was limited to daily or nearly daily basis over the last year. This is a strict definition with individuals using medication 3-4 times per week (and less frequently) not being accounted for.

The list of possible confounding factors most likely did not capture all possible confounding effects from chronic somatic or psychiatric conditions, and there might be other relevant risk factors for mortality not assessed in the current study. Self-report instruments are prone to error and residual confounding cannot therefore be ruled out. Screening for psychiatric morbidity was limited to symptoms of anxiety and depression. In addition, some of the confounders were operationalized in a rather crude way. For example, the employed cut-off for alcohol consumption was based on weekly instead of daily intake. This puts some limitation on the adjustment for confounders concerning the association between insomnia and mortality. Also, the number of deaths was limited to 160, which prevented us from conducting further sub-analyses, for example on the interaction between insomnia and OSA and use of hypnotic medication, and also precluded us from investigation of the relationship between insomnia and specific causes of death. The small number of deaths in the insomnia group also represents a problem with regards to statistical power. A related issue concerns the long list of adjustment variables that were used in the regression analyses. Given the modest sample size, it is not unproblematic to include so many confounders, which may distort some of the statistical findings. For example, we cannot easily explain why the fully adjusted HRs in several instances were larger than the unadjusted. For this reason, we did not conduct fully adjusted analyses when examining subgroups (interaction with gender and sleep duration). Also, as the current study included measures of both insomnia, sleep medications, sleep duration and OSA, multicollinearity could have produced biased results, but testing for this in the current study showed this not to be the case (with tolerance values larger than 0.61 and VIFs less than 1.63 for all predictor variables) [53]. This issue was also pointed to by Hublin et al. [44] emphasizing how difficult it is to fully separate risks of insomnia from those of similar conditions and disorders. Therefore, the findings, and especially those from the fully adjusted analyses, should be interpreted with caution and repeated by studies based on larger samples. Also, given the age in our study population, several risk factors that were not present at baseline (obesity, sleep apnea, diabetes, etc.) may have emerged later during the course of follow-up, which may have influenced the outcome. Finally, the participation rate was not very high (63%) and generalizations of the results must be made with caution as both mortality rates [54] and symptom levels [55] are higher among non-participants.

There are several strengths of this study that deserves mention. First, the design included complete follow-up through the nationwide Cause of Death Registry, made possible through linkage using the personal identification number unique to each Norwegian resident. Also, the comprehensive list of confounders, including other sleep variables, allowed investigation of the unique effect of insomnia. In addition, the study had sufficient statistical power to conduct sub-analyses of the interaction effects of insomnia and gender and sleep duration.

## Conclusion

In conclusion, insomnia was found to be a strong risk factor for death among middle-aged adults over 13-15 years follow-up, even after adjusting for potential confounders, and the results suggest the risk is stronger in, if not confined to, males and insomniacs with short sleep duration. The number of deaths observed in this study was low, and challenges statistical power, particularly in the multivariate models and subgroup analyses. As the findings were stronger compared to those reported elsewhere, they should be interpreted with caution and repeated in larger samples where the rates of outcomes are higher. Nevertheless, given the high prevalence of insomnia in the general population and the range of negative outcomes repeatedly linked with insomnia, these results should, if nothing else, support public health policy advances in prevention and low-threshold interventions for insomnia.

## Competing interests

The authors declare that they have no competing interests.

## Authors’ contributions

Authors BS and SO were responsible for conception and design of the study, and BS conducted the statistical analysis and drafted the manuscript. Author GT were involved in acquisition of data and obtained funding for the HUSK study. Authors SP, NG, BB, PS, GT, SO and RU gave critical revision of the manuscript for important intellectual content. Authors BS and SO had full access to all the data in the study and takes responsibility for the integrity of the data and the accuracy of the data analysis. All authors read and approved the final manuscript.

## Pre-publication history

The pre-publication history for this paper can be accessed here:

http://www.biomedcentral.com/1471-2458/14/720/prepub
